# Marrow tuberculosis without granuloma formation in a patient with human immunodeficiency virus infection

**DOI:** 10.1002/jha2.755

**Published:** 2023-07-06

**Authors:** Wing Kit Lam, Murphy Ka Hei Sun, Harry Ka Ngai Lau, Alice Ching Ching Wong, Sze Fai Yip

**Affiliations:** ^1^ Department of Clinical Pathology Tuen Mun Hospital Hong Kong Hong Kong; ^2^ Department of Anatomical and Cellular Pathology Prince of Wales Hospital Hong Kong Hong Kong; ^3^ Present address: Department of Medicine North District Hospital Hong Kong

**Keywords:** bone marrow pathology, HIV, infection

1

A 29‐year‐old male with unremarkable past health presented with fever of unknown origin, vomiting, diarrhea, epigastric pain, mild anemia, and thrombocytosis (hemoglobin 12.2 g/dL, platelet 415 × 10^9^/L). Human immunodeficiency virus (HIV) serology was positive with a CD4^+^ T‐cell count of <10 cells/μL. Chest radiograph was unremarkable. Positron emission tomography/computed tomography showed multiple hypermetabolic matted lymph nodes with cystic and necrotic changes in the abdomen and pelvis with diffuse marrow uptake, with differential diagnoses of multiple nodal metastases, tuberculous lymphadenitis, and lymphoma to be considered. The patient developed cardiac arrest requiring active resuscitation and intensive care. Both marrow aspirate and trephine biopsy showed mildly hypocellular marrow with serous degenerative changes, focal prominence of plasma cells and increased pyknotic megakaryocytes, characteristic of HIV infection (Figure 1). There was no granuloma formation. With Ziehl‐Neelsen stain, acid‐fast bacilli (AFB) were demonstrated (Figure 2). Both the peripheral blood and bone marrow cultures were negative for *Mycobacterium* species. The sputum smear for AFB was negative and sputum culture was positive for *Mycobacterium tuberculosis*. There is no evidence of multidrug resistance or extensive drug resistance upon susceptibility testing. Polymerase chain reaction for *Mycobacterium tuberculosis* on the trephine biopsy specimen was positive. The patient eventually succumbed 5 weeks after admission despite maximal support and antituberculous treatment.

**FIGURE 1:**
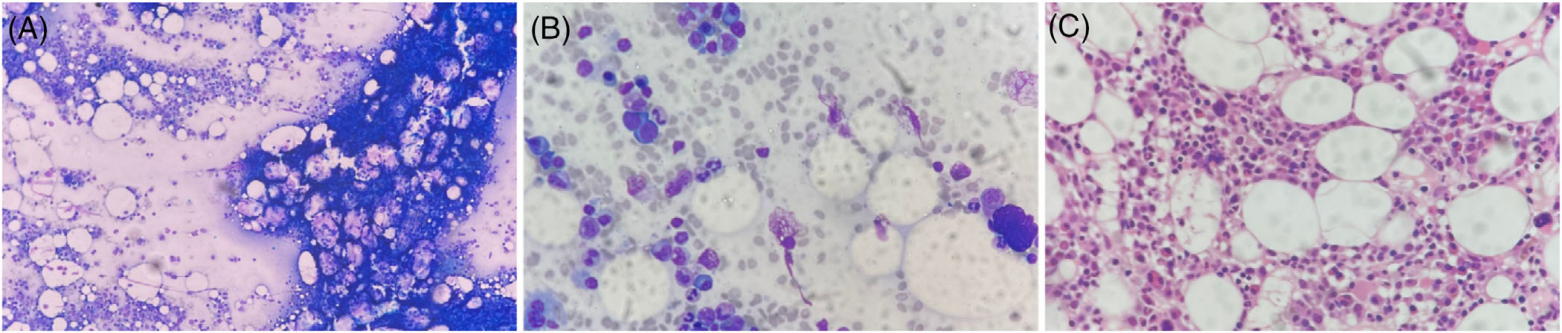
The light microscopy of the bone marrow aspirate and trephine biopsy in a patient showing HIV‐related marrow changes without granuloma formation. The bone marrow aspirate shows (a) mildly hypocellular marrow with serous degenerative changes, (b) focal prominence of plasma cells and increased pyknotic megakaryocytes. (c) The trephine biopsy also showed prominence of plasma cells and increased pyknotic megakaryocytes.

**FIGURE 2:**
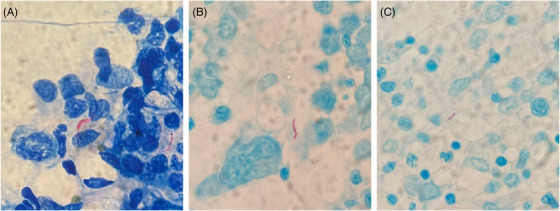
Ziehl‐Neelsen stain performed on the (a) marrow aspirate and (b, c) trephine biopsy. A few acid‐fast bacilli were seen upon Ziehl‐Neelsen stain.

The incidence of tuberculosis was previously reported to be 6.5 per 100‐person‐years of HIV patients under observation [1]. The two pathogens could potentiate one another and accelerate the deterioration of immunological functions [2]. Advanced World Health Organization clinical disease stage (III and IV), bedridden status, baseline opportunistic infections, low hemoglobin level, and not taking Isonized Preventive Therapy were risk factors for developing tuberculosis in HIV patients [1]. Previous study in India showed that 40% of HIV patients were clinically diagnosed of tuberculosis [3]. Of these, 12.86% of the bone marrow aspirates were positive for AFB. This highlighted that tuberculosis is not uncommon in HIV patients in regions with high prevalence of tuberculosis. In our locality, tuberculosis is a notifiable disease. For staff contacts, counseling would be offered and chest X‐ray if necessary. Sputum samples should be collected for those with compatible symptoms. Screening for latent tuberculous infections is still controversial in the local setting given the high prevalence of tuberculosis (about 4500 notified cases per year in Hong Kong). Marrow tuberculosis has a high mortality rate even with antituberculous treatment [4]. The typical granulomatous inflammation in marrow tuberculosis could be deceptively absent, as in this patient, suggesting the need to screen for marrow tuberculosis in HIV patients.

## AUTHOR CONTRIBUTIONS

All authors wrote the main manuscript text and LWK prepared Figures 1 and 2. All authors reviewed the manuscript.

## CONFLICT OF INTEREST STATEMENT

The authors declare that they have no known competing financial interests or personal relationships that could have appeared to influence the work reported in this paper.

## FUNDING

The author received no financial support for the research, authorship, or publication of this article.

## ETHICS STATEMENT

The information presented in this manuscript is deidentified, and there is minimal risk to the patient's privacy or confidentiality. Ethics approval was not required by our institution for preparation of this manuscript. Patient consent was not obtained. No material from other sources is included in this manuscript.

## Data Availability

Data sharing not applicable to this article as no datasets were generated or analyzed during the current study.

## References

[1] Temesgen B , Kibret GD , Alamirew NM , Melkamu MW , Hibstie YT , Petrucka P , et al. Incidence and predictors of tuberculosis among HIV‐positive adults on antiretroviral therapy at Debre Markos referral hospital, Northwest Ethiopia: a retrospective record review. BMC Public Health. 2019 Nov 27;19(1):1566. 31771552 10.1186/s12889-019-7912-9 PMC6880633

[2] Bruchfeld J , Correia‐Neves M , Källenius G . Tuberculosis and HIV coinfection. Cold Spring Harb Perspect Med. 2015 Feb 26;5(7):a017871. 25722472 10.1101/cshperspect.a017871 PMC4484961

[3] Khandekar MM , Deshmukh SD , Holla VV , Rane SR , Kakrani AL , Sangale SA , et al. Profile of bone marrow examination in HIV/AIDS patients to detect opportunistic infections, especially tuberculosis. Indian J Pathol Microbiol. 2005 Jan;48(1):7–12. 16758774

[4] Hakawi AM , Alrajhi AA . Tuberculosis of the bone marrow: clinico‐pathological study of 22 cases from Saudi Arabia. Int J Tuberc Lung Dis. 2006;10(9):1041–4. 16964798

